# Use of Staged Molecular Analysis to Determine Causes of Unexplained Central Nervous System Infections

**DOI:** 10.3201/eid1909.130474

**Published:** 2013-09

**Authors:** Chien-Chin Hsu, Rafal Tokarz, Thomas Briese, Hung-Chin Tsai, Phenix-Lan Quan, W. Ian Lipkin

**Affiliations:** Columbia University, New York, New York, USA (C.-C. Hsu, R. Tokarz, T. Briese, P.-L. Quan, W.I. Lipkin);; Southern Taiwan University of Science and Technology, Tainan, Taiwan (C.-C. Hsu);; Chi-Mei Medical Center, Tainan (C.-C. Hsu);; National Yang-Ming University Kaohsiung, Taiwan (H.-C. Tsai);; Kaohsiung Veterans General Hospital, Kaohsiung (H.-C. Tsai)

**Keywords:** CNS infections, meningitis, encephalitis, MassTag PCR, 16S rRNA PCR, microarray, pyrosequencing, viruses, bacteria

## Abstract

No agent is implicated in most central nervous system (CNS) infections. To investigate cerebrospinal fluid samples from patients with CNS infections of unknown cause in 1 hospital in Taiwan, we used a staged molecular approach, incorporating techniques including multiplex MassTag PCR, 16S rRNA PCR, DNA microarray, and high-throughput pyrosequencing. We determined the infectious agent for 31 (24%) of 131 previously negative samples. Candidate pathogens were identified for 25 (27%) of 94 unexplained meningitis cases and 6 (16%) of 37 unexplained encephalitis cases. Epstein-Barr virus (18 infections) accounted for most of the identified agents in unexplained meningitis cases, followed by *Escherichia coli* (5), enterovirus (2), human herpesvirus 2 (1), and *Mycobacterium tuberculosis*. Herpesviruses were identified in samples from patients with unexplained encephalitis cases, including varicella-zoster virus (3 infections), human herpesvirus 1 (2), and cytomegalovirus (1). Our study confirms the power of multiplex MassTag PCR as a rapid diagnostic tool for identifying pathogens causing unexplained CNS infections.

Central nervous system (CNS) infections pose a diagnostic challenge because clinical manifestations are not typically pathognomonic for specific pathogens, and a wide range of agents can be causative. An infectious cause of encephalitis is determined for <40%–70% of cases worldwide (*1*–*5*). Culture is of limited use, particularly for viral infections. In recent studies, only 1.9% of cerebrospinal fluid (CSF) viral cultures were positive (*3*), and <0.1% of CSF cultures recovered viruses other than enteroviruses or herpesviruses (*6*).

PCR enables sensitive detection of microbial nucleic acids in clinical samples, which may be useful for identifying pathogens that are nonviable, uncultivable, or fastidious. MassTag PCR is a multiplex platform that enables inexpensive, sensitive, and simultaneous detection of multiple pathogens (*7*–*10*). Originally implemented for differential diagnosis of respiratory tract infections (*7*), MassTag PCR has been expanded to several syndrome-based panels for differential diagnosis of hemorrhagic fever and for detection of tick-borne pathogens (*9*,*11*).

Amplification and sequencing of the 16S ribosomal RNA (rRNA) gene is a well-established technique for identifying bacterial pathogens (*12*). Broad-range 16S rRNA PCR with subsequent sequencing is superior to bacterial culture for diagnosing bacterial meningitis, with a sensitivity of 86% and a specificity of 97% (*13*). It is particularly useful for slow-growing bacteria (e.g., *Mycobacterium tuberculosis*) and for diagnosis of cases that remain culture-negative as a result of antimicrobial drug treatment before lumbar puncture.

Microarray technology also has been applied to the detection and identification of infectious diseases (*10*,*14*) and has the potential to test for virtually all known viruses, bacteria, fungi, and parasites (*15*).The advent of high-throughput sequencing affords unique opportunities for pathogen surveillance and discovery with regard to CNS infections. We have successfully used high-throughput sequencing to identify causative agents of disease for patients with transplant-associated encephalopathy (*16*) and encephalitis associated with agammaglobulinemia (*17*).

Our staged molecular approach used complementary tools for pathogen detection and discovery that include syndrome-based multiplex PCRs, DNA microarray, and high-throughput sequencing (*18*). We report the results of our investigation of CNS infections of unknown cause in 1 hospital in Taiwan, which we conducted by using MassTag PCR, 16S rRNA PCR, DNA microarray, and high-throughput sequencing.

## Methods

### Patients

Meningitis was diagnosed by clinical features of fever (>38°C), headache, nuchal rigidity, abnormal CSF profile (CSF protein >45 mg/dL [reference 15–45 mg/dL] and leukocyte >5/μL [reference 0–5/μL]), and/or meningeal involvement noted by brain magnetic resonance imaging (MRI). Encephalitis was diagnosed when there was evidence of brain parenchyma involvement (e.g., paralysis or focal or generalized seizure) and when the finding of brain MRI or electroencephalogram (EEG) was compatible with encephalitis. CSF samples from meningitis or encephalitis patients with noninfectious diagnosis, including traumatic, metabolic, malignant, vascular, surgical, hypoxic, and toxic causes, were excluded.

### CSF Samples

A total of 212 CSF samples from 212 patients (165 meningitis and 47 encephalitis cases) were obtained during 2006–2008 in Kaohsiung Veterans General Hospital, a tertiary care hospital in southern Taiwan. Tests conducted in the hospital included bacterial and viral culture, cryptococcal antigen test, VDRL (Venereal Disease Research Laboratory) test and human herpesvirus (HHV) 1 PCR. Pathogens were identified for 71 meningitis patients and included*Cryptococcus neoformans* (36 cases), *M. tuberculosis* (18), *Klebsiella pneumonia* (7), enterovirus (4), *Streptococous pneumoniae* (2), *Listeria monocytogenes* (2), and *S. viridians* (2). Pathogens were identified for 6 encephalitis patients and includedJapanese encephalitis virus (3 cases) and herpesviruses (3). The remaining 131 patients with unexplained meningitis or encephalitis with CSF pleocytosis and without a definitive diagnosis were included in this study. CSF samples were stored at –70°C until analysis.

### Sample Preparation

Total nucleic acids were extracted from 250-μL CSF samples by the NucliSENS Easy Mag Extraction Method (bioMérieux, Marcy l’Etoile, France) and eluted in 35 μL nuclease-free water. We performed reverse transcription (RT) PCR for human β-actin gene to ensure the quality of the extracted DNA and RNA. For RT, total nucleic acids were enriched for RNA with a DNase I treatment (DNA-free; Ambion, Austin, TX, USA). RT was performed by using random hexamers with the Invitrogen Superscript II Kit (Invitrogen, Carlsbad, CA, USA).

### MassTag PCR

MassTag PCR is a platform that enables inexpensive, sensitive, and simultaneous detection of multiple pathogens (*7*–*10*). In this study, we used a MassTag PCR platform that targets 29 known CNS pathogens, including 21 viruses, 5 bacteria, 2 fungi, and 1 parasite. Detailed gene targets and primer sequences are listed in Tables 1 and 2. We first tested unmodified (i.e., untagged) primer sets in singleplex reactions for specificity with the cognate and negative control targets. All primers were then used in multiplex PCR to test for interference. Primers yielding a single and specific PCR product band on agarose gel electrophoresis were taken forward for synthesis and conjugation with tags of varying mass. These primers were included in the panel. Target DNA standards for panel development were cloned into pCR2.1 TOPO (Invitrogen) by PCR amplification of pathogen DNA or cDNA templates (RNA viral targets). A sensitivity assay was performed with 10-fold dilutions of linearized plasmid against a background of 5 ng/μL of human placental DNA.

**Table 1:** Primers for MassTag central nervous system infections panel, RNA pathogens

**Pathogen**	**Target gene**	Primer sequence, 5′↔3′*	**Mass code**
**Eastern equine encephalitis virus**	E1	Fwd: ACACTAAATTCACCCTAGTTCGAT	
		Rev: GTGTATAAAATTACTTAGGAGCAGCATTATG	383/650
**Nipah/Hendra virus**	Phos	Fwd: GGGGGAATGYCTAAGRATGATG	
		Rev: TCCGGTACATTCTCCTCCATG	519/566
**Japanese encephalitis virus**	NS5	Fwd: TCAACCTAGGGAGCGGAACA	
		Rev: GGCTGAGCCAGTAGCCTTCA	582/698
**Parechovirus**	5UTR	Fwd: ACACTAGTTGTAAGGCCCACGAA	
		Rev: GGTBTGGCCCACTAGACGTTTT	690/606
**Powassan virus**	NS5	Fwd: CATCCGACCATGCACCTAGA	
		Rev: CCAAAGTGAGGATGTGTACCAAAG	622/375
**La Crosse virus**	S	Fwd: CTCAACCTTGCTGCAGTTAGGA	
		Rev: CCACCTGCCACTCTCCAAA	686/590
**Lymphocytic choriomeningitis virus**	pol	Fwd: CCACTYTTGTCTGCACTGTCTAT Rev: CTTTTTGATGCGCAATGGAT	
			614/654
**St. Louis encephalitis virus**	NS5	Fwd: CATTTGTTCAGCTGTCCCAGTC	
		Rev: CTCACCCTTCCCATGAATTGA	658/423
**Enteroviruses**	5UTR	Fwd: TCCTCCGGCCCCTGAATGCGGCTAATCC	
		Rev: GAAACACGGWCACCAAAGTASTCG	495/702
**West Nile virus**	DF3	Fwd: CCACCGGAAGTTGAGTAGACG	
		Rev: GCTTTGTTCACCCAGTCCTCCT	499/539
**Western equine encephalitis virus**	E1	Fwd: ACATCGAGCCCACAAGCA	
		Rev: GCATAGAGCTGCAGACCAACAC	598/678
**Venezuelan equine encephalitis virus**	E1	Fwd: CTACGCGCCACTCCCTATCA	
		Rev: TGGCAGGTGACGTACTCCAA	602/646
**Rabies virus**	N	Fwd: GGGTTYATAAAVCAGATWAATCTCAC	
		Rev: GAAGTGRATGAAATARGAGTGAGG	475/558
**Influenza A virus**	M	Fwd: CATGGAATGGCTAAAGACAAGACC	
		Rev: AAGTGCACCAGCAGAATAACTGAG	618/690

*Fwd, forward primer; Rev, reverse primer.

**Table 2:** Primers for MassTag CNS infections panel: DNA pathogens

Pathogen	Target gene	Primer sequence, 5′→3*	Mass code
Virus			
Adenoviruses	Hexon	Fwd: CCCMTTYAACCACCACCG	
		Rev: ACATCCTTBCKGAAGTTCCA	503/630
Cytomegalovirus	Pol	Fwd: CATGCGCGAGTGTCAAGAC	
		Rev: ACTTTGAGYGCCATCTGTTCCT	610/626
Epstein-Barr virus	EBER	Fwd: AAACCTCAGGACCTACGCTGC	
		Rev: AGACACCGTCCTCACCAC	570/463
Varicella-zoster virus	Gp 31	Fwd: CCGATTCTGGATTTTCGTTGTT	
		Rev: AAAGTCGATTTCCCCCCAAA	471/515
Human herpesvirus 6	U7	Fwd: AAAATTTCTCACGCCGGTATTC	
		Rev: CCTGCAGACCGTTCGTCAA	357/718
Human herpesvirus 1	Gp C1	Fwd: GATGCCGGTTTCGGAATTC	
		Rev: CCCATGGAGTAACGCCATATCT	706/666
Human herpesvirus 2	UL3	Fwd: GGTCCCCTCTGCGTTTACTA	
		Rev: TCGACTCTATGGGCGTCGTA	527/642
Bacterium			
* Haemophilus influenzae*	hgbC	Fwd: CGCTGGAAAGAGAACAAGCAA	
		Rev: TTTCAGCTTGACGTAATCCATC	726/734
* Streptococcus pneumoniae*	plysin	Fwd: GACTCCTAAGGCTTGGGACAGAAAT	
		Rev: TTCATAAACCGTACGCCACCATTC	694/714
* Neisseria meningitides*	ctrA	Fwd: TTCTGATGCGCGTGGTGTGT	
		Rev: CGCATCAGCCATATTCACACGA	439/730
* Leptospira interrogans*	flaB	Fwd: GATCATGAAGCAGAGRGCGGATATG	
		Rev: CCATATCGGCGTCYCGAATTC	634/383
* Mycobacterium tuberculosis*	pncA	Fwd: ACGTCAGGCCCACGACATTGA	
		Rev: CCTGGGCAAGCTGAACCTCGAA	395/475
Parasite: *Toxoplasma gondii*	B1	Fwd: GAAGAGATCCAGCAGATCTCGT	
		Rev: TGAGAGGAGGCAGCACAAG	548/562
Fungus			
* Candida albicans*	CaAG	Fwd: ACCAGTAGGAGTACAACGAACAGGAA	
		Rev: ATTTCATTGAATATTGGTGTGGTTCA	602/670
* Cryptococcus neoformans*	cap59	Fwd: GCGAGGCAGCACAAGTACTT	
		Rev: TTGTCTGGTCGTTGGAMCCGTT	650/638

*Fwd, forward primer; Rev, reverse primer.

For the DNA agent test panel, total nucleic acids (4 μL) were added to MassTag PCR reaction mixes containing the DNA pathogens primer mix and were amplified by a standard cycling protocol, as follows: denaturation at 94°C for 20 s, annealing with a temperature reduction in 1°C increments from 65°C to 51°C during the first 14 cycles and then continuing for 35 cycles at 50°C for 20 s, then extension 72°C for 30 s in an MJ PTC200 thermal cycler (MJ Research, Waltham, MA, USA). For the RNA agent test panel, 4 μL of RT products were added to MassTag PCR reactions containing the RNA pathogens primer mix and were amplified by the same protocol.

PCR products were purified by using QIAquick 96 PCR purification cartridges (QIAGEN, Hilden, Germany) to remove unincorporated primers before the tags were released from PCR products by UV irradiation in a flow cell. Tags were analyzed in a single quadrupole mass spectrometer by using positive-mode atmospheric-pressure chemical ionization (Agilent Technologies, Wilmington, DE, USA) (*7*). The identity of a pathogen in the CSF was determined by the presence of its cognate tags. The negative controls consisted of irrelevant DNA. A positive sample was defined as a sample in which an agent was detected with both tags giving signal above a threshold defined as the 95th percentile cut point of the negative control distribution, and positivity was assessed by using the interquartile ranges of this distribution.

### 16S rRNA Gene PCR

For samples that were negative by MassTag PCR, we performed broad-range bacterial 16S rRNA PCRs to detect bacterial sequences not addressed in the MassTag panel. To minimize potential for spurious amplification of 16S rRNA sequences in reagents, we adopted the DNase I (Ambion DNA-free; Austin, TX, USA) pretreatment as described in Heininger et al. (*19*) to the PCR master mix, including the incubation of AmpliTaq Gold polymerase (Applied Biosystems, Foster City, CA, USA), MgCl_2_, PCR 10× buffer and dNTPs with 0.1 IU DNase I for 30 min at 37°C, then for 10 min at 95°C. Primers and templates were added subsequently. 16S rRNA was amplified (899-bp length) with 2 universal primers, 8UA (5′-AGAGTTTGATCCTGGCTCAG-3′) and 907B (5′-CCGTCAATTCMTTTAGTTT-3′) (*20*). Cycling conditions were 95°C for 5 min, followed by 35 cycles at 95°C for 30 s, 45°C for 30 s, and 72°C for 60 s, with a final elongation at 72°C for 5 min.

### DNA Microarray

#### Sample Preparation

RNA was enriched from total nucleic acids by DNase I treatment (DNA-free; Ambion, Austin, TX, USA) to eliminate human chromosomal DNA. First-strand RT was initiated by using Superscript II (Invitrogen) with a random octamer linked to a specific primer sequence (5′-GTT TCC CAG TAG GTC TCN NNN NNN N-3′) (*14*). After digestion with RNase H (Invitrogen), the cDNA was amplified by using a 1:9 mixture of the random octamer–linked primer and the specific primer sequence (5′-CGC CGT TTC CCA GTA GGT CTC-3′). A low annealing temperature (25°C) was used for the initial PCR amplification cycle; a stringent annealing temperature (55°C) was used thereafter to favor priming through the specific sequence (*15*). The products of this first PCR (18 cycles) were subsequently labeled by a primer that included the specific primer sequence linked to a capture sequence (49 cycles) that, after array hybridization, permitted the labeling through binding of 3 DNA dendrimers containing >300 fluorescent reporter molecules (Genisphere Inc., Hatfield, PA, USA).

#### Microarray Hybridization and Processing

Hybridization was performed by adding 30 μL of sodium dodecyl sulfate-based hybridization buffer (Genisphere Inc.) to the products of the second-labeling PCR, heating for 10 min at 80°C, and transferring the solution to the Greenchip Vir microarray (*15*) for hybridization for 16 hours at 65°C. The arrays were washed with 6X SSC (0.15 M NaCL plus 0.015 M sodium citrate), 0.005% Triton X-100, and 0.1× SSC-0.005% Triton X-100 for 10 min at room temperature. Cy3 3DNA dendrimers (Genisphere Inc.) were then added for a secondary hybridization at 65°C for 1 hour before a final wash (*15*).

#### Microarray Scanning and Analysis

The microarrays were scanned by the NimbleGen MS 200 Microarray scanner (Roche NimbleGen, Madison, WI, USA), and analyzed with GreeneLAMP software (version 2.0) (*15*). A ranked list of candidate organisms was identified by linking probe sequences with positive signal on the array to the matching sequences in a viral sequence database; each of which corresponded to a taxonomic identifier (NCBI Taxon ID, TaxID). The individual TaxIDs were finally mapped to nodes in a phylogenetic tree that was constructed in accordance with data from the International Committee on Taxonomy of Viruses.

#### Pyrosequencing

The 100 samples for which no agent was identified after MassTag PCR and array analysis were pooled and analyzed by high-throughput pyrosequencing. The same sample preparation protocol as for array analysis up to the second-labeling PCR was used to amplify material for high-throughput sequencing analysis. Products >70 bp long were purified by using a MinElute kit (QIAGEN, Valencia, CA, USA). Purified products were pooled and sequenced on the GSL FLX platform (454 Life Sciences, Branford, CT, USA). Raw sequence reads were trimmed to remove sequences primer and highly repetitive sequences. In a second step, reads were clustered and assembled into contiguous fragments for comparison with the GenBank database by the BLAST algorithm (http://blast.ncbi.nlm.nih.gov/Blast.cgi) by using nucleotide and deduced amino acid sequence (*21*).

#### Confirmation of Pathogens 

We confirmed the identify of pathogens using several methods. All pathogens indicated by MassTag PCR, microarray, and pyrosequencing were confirmed by targeted singleplex PCR amplification, sequencing of the products, and BLAST analysis of the obtained sequences.

#### Serology Analysis

For Epstein-Barr virus (EBV) DNA–positive CSF samples, we measured EBV antibodies by using the commercial kit Euroimmun (Medizinische Labordiagnostika, Lübeck, Germany), which tests for IgG and IgM against EBV viral capsid antigen (VCA), EBV early antigen, and nuclear antigen. Results were evaluated semiquantitatively by calculating a ratio of the extinction value of the control or patient samples over the extinction value of calibrator (ratio <0.8: negative; ratio >1.1: positive).

## Results

A total of 131 CSF samples were collected during 2006–2008 from adults with unexplained meningitis or encephalitis. The patients in this study were 18–86 years of age (median 39 years); the male-to-female ratio was 2.4:1. Fourteen percent of patients had diabetes, and 8% were HIV positive. In addition to fever and headache, mental status was abnormal for 22% of patients; 2% had paralysis/seizure.

Two patients with unexplained encephalitis died. Samples were analyzed by applying a staged molecular approach, including multiplex MassTag PCR, 16S rRNA-gene PCR, DNA microarray, and high-throughput sequencing (Figure). We identified 31 (24%) pathogens in 131 CSF samples from patients with meningitis and encephalitis of unknown cause. Candidate pathogens were identified for 25 (27%) of 94 patients with unexplained meningitis and 6 (16%) of 37 with unexplained encephalitis (Table 3). EBV (16 cases) accounted for most of the identified pathogens in unexplained meningitis cases, followed by *Escherichia coli* (5 cases), enterovirus (2), HHV-2 (1), and *M. tuberculosis* (1). Herpesviruses were identified for unexplained encephalitis cases and includedvaricella-zoster virus (3 cases), HHV-1 (2), and cytomegalovirus (1). We also tested 3 CSF samples from patients with noninfectious CNS disease as negative controls; no agents were identified in these samples.

**Figure:**
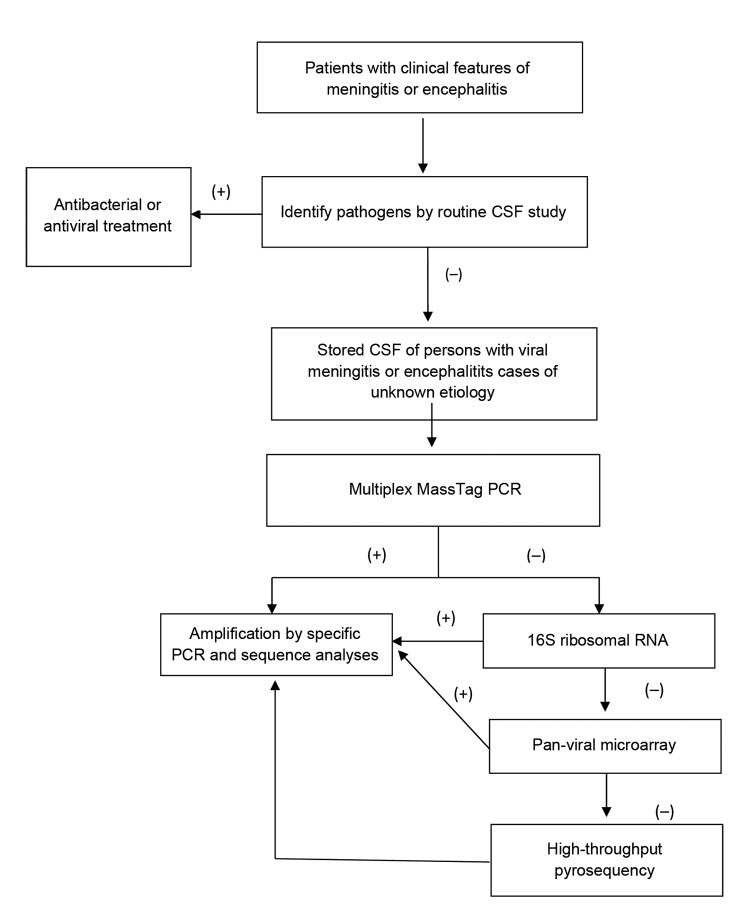
Molecular approaches used to investigate central nervous system infections of unknown cause. Routine study of CSF comprised chemistry, direct microbial examinations, antigen tests, and bacterial and viral cultures. CSF, cerebrospinal fluid; +, positive; –, negative.

**Table 3:** Pathogens identified by a staged molecular approach

**Pathogen**	**No. (%) cases**			**No. HIV positive**	**Molecular method**
	Total	Meningitis	Encephalitis		
**Virus**					
Enterovirus*	2 (6)	2 (8)	0	0	MassTag PCR
** Human herpesvirus 1**	2 (6)	0	2 (33)	0	MassTag PCR/microarray†
** Human herpesvirus 2**	1 (3)	1 (4)	0	0	MassTag PCR
** Varicella-zoster virus**	3 (10)	0	3 (50)	0	MassTag PCR
** Epstein-Barr virus**	16 (52)	16 (64)	0	9	MassTag PCR
** Cytomegalovirus**	1 (3)	0	1 (17)	0	MassTag PCR
**Bacteria**					
*** Mycobacterium tuberculosis***	1 (3)	1 (4)	0	0	MassTag PCR
*** Escherichia coli***	5 (16)	5 (20)	0	1	16S rRNA PCR
**Total**	31 (100)	25 (100)	6 (100)	10	

*Sequences of enteroviruses showed 1 infection each of echovirus 11 and echovirus 30. **†For 1 case, human herpesvirus 1 was identified by MassTag PCR; in the other, by DNA microarray.**

Five bacterial pathogens were identified by 16S rRNA PCR (*E. coli*, 5 patients), and 1 HHV-1 was identified by microarray analysis. We pursued unbiased high-throughput pyrosequencing of RNA from CSF by pooling the remaining negative samples (screened previously by MassTag PCR and microarrays); however, no specific pathogen was identified by pyrosequencing. All positive samples detected by MassTag PCR, 16S rRNA PCR, and microarray were verified by targeted singleplex PCR amplification and sequencing.

Of 16 EBV-positive patients, 9 (56%) were HIV infected. To differentiate EBV latent infection from lytic infection, we further measured EBV-specific antibodies in EBV DNA–positive CSF samples; in 8 cases, paired CSF and serum samples were available for analysis (Table 4). IgG against EBV VCA was found in 11 (69%) of 16 EBV DNA–positive CSF samples; however, none of the CSF samples were positive for VCA IgM, and only 1 CSF sample was positive for both EBV early antigen and nuclear antigen, indicating current or recent EBV infection. All 8 serum samples, which were paired with 8 of the CSF samples, were positive for VCA IgG and EBV nuclear antigen but negative for VCA IgM and EBV early antigen, suggesting that latent EBV infections existed in those patients.

**Table 4:** EBV antibody in CSF and serum of patients identified with EBV infections by MassTag PCR*

**Case**	**CSF**					**Serum**			
	VCA IgM	VCA IgG	EBV EA	EBV NA		VCA IgM	VCA IgG	EBV EA	EBV NA
**1**	–	–	–	–		–	+	–	+
**2**	–	–	–	–		–	+	–	+
**3**	–	–	–	–		ND	ND	ND	ND
**4**	–	+	–	–		ND	ND	ND	ND
**5**	–	+	+	+		ND	ND	ND	ND
**6**	–	+	–	–		–	+	–	+
**7**	–	+	–	–		–	+	–	+
**8**	–	+	–	–		ND	ND	ND	ND
**9**	–	+	–	–		ND	ND	ND	ND
**10**	–	+	–	–		–	+	–	+
**11**	–	–	–	–		–	+	–	+
**12**	–	+	–	–		–	+	–	+
**13**	–	+	–	–		ND	ND	ND	ND
**14**	–	+	–	–		ND	ND	ND	ND
**15**	–	+	–	–		ND	ND	ND	ND
**16**	–	–	–	–		–	+	–	+

*EBV antibody was measured by using commercial kit Euroimmun (Medizinische Labordiagnostika, Lübeck, Germany). Results were evaluated semiquantitatively by calculating a ratio of the extinction value of the control or patient samples over the extinction value of calibrator. Ratio <0.8: negative; ratio >1.1: positive. EBV, Epstein-Barr virus; CSF, cerebrospinal fluid; VCA, viral capsid antigen; EBV NA, EBV nuclear antigen indicating past EBV infections; EBV EA, EBV early antigen, indicating current or recent EBV infection; –, negative; +, positive; ND, not done.

## Discussion

We have described results of a comprehensive, staged molecular analysis of unexplained encephalitis and meningitis conducted by using CFS samples obtained over a 3-year period in a major referral hospital in Taiwan. Our investigation confirmed the presence of microbial sequences in 31 (24%) of 131 CSF samples; 25 were identified by MassTag PCR.

We detected EBV in 16 samples. EBV is a ubiquitous HHV that infects 90% of adults worldwide (*22*). In Taiwan, too, most persons are infected with EBV in early childhood (*23*). VCA was detected in all EBV-positive CSF samples. This finding is consistent with EBV-related CNS disease. In a study of 5 patients with mononucleosis, all had EBV DNA and EBV-specific antibodies in the CSF during the acute phase of disease in association with neurologic manifestations but not during convalescence (*24*). In contrast, in the same study, EBV DNA and EBV antibodies were not detected in the CSF of 17 EBV-seropositive patients with other CNS infections, such as mumps, meningitis, and rubella encephalitis (*24*).

It could be argued that any inflammatory process has the potential to carry EBV into the CSF as a passenger in infiltrating lymphocytes; however, PCR results for EBV in CSF are rarely positive for patients with other CNS infections (*25*). One study showed that only 11 of 2,233 specimens from 2,162 patients were EBV positive by PCR (*26*). In another study, EBV was not detected by PCR of CSF from patients with bacterial meningitis, despite the presence of more lymphocytes/monocytes in the CSF of control patients with bacterial meningitis than in the CSF of the EBV-positive patients (*27*). In accord with these results, we did not detect EBV in the CSF of patients with CNS infections of bacterial origin, such as *M. tuberculosis* and *E. coli*.

All of the 16 EBV-positive patients in our study had meningitis; none had encephalitis. EBV is an uncommon cause of meningitis (0.9%) (*28*) and of encephalitis (2.3%) (*29*). However, given the data reported here, EBV-associated CNS infections in immunocompromised patients may be underestimated. In our study, 56% of EBV-positive patients were HIV infected. EBV has been reported to be associated with primary CNS lymphomas in HIV-infected persons (*30*,*31*), and EBV DNA can be detected in the CSF of 80%–100% of HIV-infected patients with primary CNS lymphomas (*32*–*34*); however, EBV also may be detected in the CSF of 12% of HIV-infected patients with neurologic symptoms but without lymphoma (*35*). None of the EBV-positive patients in our study had evidence of CNS lymphoma by contrast computed tomography or MRI of the brain. Because EBV is a ubiquitous virus, the incidence of CNS infections caused by EBV may be underestimated. Our results suggest that EBV should be considered for all patients with CNS infections, especially in immunocompromised patients.

We used 16S rRNA gene amplification to screen for any bacteria in CSF before analysis by microarray and pyrosequencing. *E. coli* was detected in 5 samples that had been culture negative in previous analysis, most likely because of prior treatment with antibacterial drugs, which can confound the results of bacterial culture. Indeed, CSF can rapidly become sterile for bacteria after antibacterial treatment, with meningococci and pneumococci becoming noncultivable within 2 and 4 hours, respectively (*36*). *E. coli* meningitis is a common cause of neonatal meningitis (*37*), but it is a rare cause of acute bacterial meningitis in adults (*38*). In Taiwan, *E. coli* meningitis has been reported in adults with chronic underlying conditions (*39*). Among 5 patients identified with *E. coli* in our study, 3 had underlying clinical conditions, including pulmonary tuberculosis (2 patients) and HIV infection (1).

In our study, most of the candidate pathogens were identified by the multiplex MassTag PCR and 16S rRNA PCR. Although this finding may raise questions about the utility of more expensive and sophisticated techniques, such as high-throughput pyrosequencing for detecting CNS infections, the unique potential of high-throughput pyrosequencing has been demonstrated by its implication of an arenavirus in transplant-associated encephalitis (*16*) and a novel astrovirus in XLA-linked agammaglobulinemia-associated encephalitis (*17*). This approach may facilitate pathogen discovery for patients with CNS infections of unexplained cause as it becomes more popular in molecular diagnostics.

Our study has some limitations. First, because samples were collected from only 1 referral hospital, our results may not reflect the case distribution of CNS infections in Taiwan but instead might represent some of the more difficult-to-diagnose cases. Second, CSF samples were stored for 3 years after collection, and nucleic acid degradation, particularly of RNA, might have occurred before laboratory analysis, which may explain the low number of RNA viruses detected. Third, we cannot rule out misclassification of encephalitis with noninfectious causes, such as the newly described immune-mediated anti-N-methyl-D-aspartate receptor encephalitis (*40*), for which we did not test.

Rapid and accurate identification of the causative agent of a CNS infection can affect clinical management of individual patients. On the scale of populations, agent identification is crucial for determining the incidence of CNS infections caused by specific agents, enabling prioritization of targets for public health intervention and to prevent outbreaks of disease.

Our study confirms the power of multiplex MassTag PCR as a rapid diagnostic tool for identifying pathogens for patients with CNS infections and shows that viral and bacterial pathogens were detected in CSF from patients with CNS infections of unidentified cause. Additionally, the staged molecular approach incorporating complementary tools may enable detection of pathogens for patients with CNS infections of previously unrecognized causes, which would otherwise be missed. This approach may aid in explaining the observed worldwide high proportion of CNS infections of unknown cause.
